# Role of insurance in determining utilization of healthcare and financial risk protection in India

**DOI:** 10.1371/journal.pone.0211793

**Published:** 2019-02-05

**Authors:** Shankar Prinja, Pankaj Bahuguna, Indrani Gupta, Samik Chowdhury, Mayur Trivedi

**Affiliations:** 1 School of Public Health, Post Graduate Institute of Medical Education and Research, Chandigarh, India; 2 Health Policy Research Unit, Institute of Economic Growth, University of Delhi Enclave, Delhi, India; 3 Indian Institute of Public Health, Gandhinagar, Gujarat, India; University of Malta Faculty of Health Sciences, MALTA

## Abstract

**Background:**

Universal health coverage has become a policy goal in most developing economies. We assess the association of health insurance (HI) schemes in general, and RSBY (National Health Insurance Scheme) in particular, on extent and pattern of healthcare utilization. Secondly, we assess the relationship of HI and RSBY on out-of-pocket (OOP) expenditures and financial risk protection (FRP).

**Methods:**

A cross-sectional study was undertaken to interview 62335 individuals among 12,134 households in 8 districts of three states in India i.e. Gujarat, Haryana and Uttar Pradesh (UP). Data on socio-demographic characteristics, assets, education, occupation, consumption expenditure, illness in last 15 days or hospitalization during last 365 days, treatment sought and its OOP expenditure was collected. We computed catastrophic health expenditures (CHE) as indicator for FRP. Hospitalization rate, choice of care provider and CHE were regressed to assess their association with insurance status and type of insurance scheme, after adjusting for other covariates.

**Results:**

Mean OOP expenditures for outpatient care among insured and uninsured were INR 961 (USD 16) and INR 840 (USD 14); and INR 32573 (USD 543) and INR 24788 (USD 413) for an episode of hospitalization respectively. The prevalence of CHE for hospitalization was 28% and 26% among the insured and uninsured population respectively. No significant association was observed in multivariate analysis between hospitalization rate, choice of care provider or CHE with insurance status or RSBY in particular.

**Conclusion:**

Health insurance in its present form does not seem to provide requisite improvement in access to care or financial risk protection.

## Introduction

Universal health coverage (UHC) has become an important stated policy goal in several developing countries [1]. UHC ensures that quality health services are accessible to all those in need, without any financial hardship. In India, aspiration of UHC has been envisaged at policy level as early as the Health Survey and Development Committee report in 1946 [2]. In 2012, a push was given through the High Level Expert Group Report, followed by its inclusion in the 12^th^ Five Year Plan [3]. More recently, another impetus has been received as a result of its inclusion in the Sustainable Development Goals for Health [4].

In terms of financing, a supply side driven approach which focussed on strengthening of health care delivery infrastructure and workforce was dominant in India till 2007. A couple of social health insurance schemes existed—*Employee State Insurance Scheme* (ESIS) and *Central Government Health Scheme* (CGHS), which had a minimal coverage [5]. In a paradigm shift to the earlier policy, several publicly financed health insurance schemes were launched at the Central and State level during the period from 2007–2010. These schemes include the *Rashtriya Swasthiya Bima Yojna* (RSBY)–now called *Rashtriya Swasthya Suraksha Yojana* (RSSY) at central level and; *Rajiv Aarogyasri* scheme (RAS) in Andhra Pradesh, *Rajiv Gandhi Jeevandayee Arogya Yojna* (RGJAY) in Maharashtra and Chief Minister’s Comprehensive Health Insurance scheme (CMCHIS) in Tamil Nadu, all at state level [6–9]. These schemes catapulted the overall coverage from about 16 million families and 75 million individuals before 2007 to 216 million individuals in 2013–14 [5, 10]. Since the source of funding for these schemes was general taxation, introduction of these schemes meant a significant allocation towards demand side financing mechanisms. RSBY alone received a cumulative allocation of INR 370 billion (USD 587 million) since 2008–09 [11].

In view of this high allocation, and emphasis being laid on these schemes as a means to achieve UHC, it is important to understand their impact. Several studies had shown an increase in healthcare utilization among those who are insured, implying an improvement in access to care [11–15]. However, other studies also report an association between the proportion of private empanelled hospitals with increased utilization rates, indicating a possibility of over-supply of health services [16–19]. As per a recent systematic review [20], 3 out of 4 impact evaluation studies done in different Indian states show no reduction in catastrophic health expenditures among insured [11, 21, 22]. One study reported a decline in out-of-pocket (OOP) payments and catastrophic health expenditures [23]. Besides the relative dearth of studies evaluating this important policy question, methodological robustness of existing studies is another issue, mainly subject to absence of an appropriate control group [24–26].

In order to fill this gap in evidence we undertook a study to assess the role of health insurance (HI) schemes in general, and RSBY in particular, in determining the extent and pattern of healthcare utilization. Secondly, we assess the effect of health insurance on OOP expenditures and financial risk protection.

## Methodology

### Data source and sampling methods

In this paper, we use data from a larger study which focuses holistically on the policy needs for universal health coverage in terms of health coverage, public expenditure and subsidies on existing health coverage, demand for health care, population covered under any insurance scheme, burden of health expenditure on households including out-of-pocket (OOP) spending, links between health expenditure and poverty and public-private dimensions of demand for care [27, 28]. A total of 62335 individuals from 12,134 households were sampled from 8 districts across three states of Gujarat, Haryana and Uttar Pradesh (UP) in India. Three states were strategically selected based on economic development and health outcomes. UP is one among the 7 EAG (Empowered Action Group) states that receives particular focus in Government programmes because of its developmental challenges. Both Haryana and Gujarat are economically advanced states with high per capita incomes. In 2012–13 the per capita incomes at current prices in Haryana, Gujarat and UP were Rs 1,22,660, Rs 96,976 and Rs 33,269 respectively [25]. In terms of health outcomes as well, Census data (in 2013) indicates that UP is doing poorly in terms of both IMR and MMR with values of 50 and 285, whereas Gujarat is doing much better (at 36 and 112 respectively) relative to Haryana (41 and 127 respectively) on both these indicators [29, 30]. Within the states, 2 districts were selected that were predominantly rural and urban respectively and one district that had a mixed residence of the population. Chota Udepur district in Gujarat was selected because of its predominantly tribal population.

A total of 10% of the rural sub-centres were randomly selected as Primary Sampling Units (PSU). Similarly in the urban area, 10% of the urban areas as enumerated for the Intensified Pulse Polio Immunization (IPPI) rounds were sampled. In each PSU area, the sample was distributed in all the villages/colonies by probability proportional to size (PPS) method. A household enumeration survey was undertaken in each village which yielded the sampling frame. Households within each PSU were then selected using systematic random sampling. Selected households were visited to inquire about an episode of illness in last 15 days or a hospitalization during last 365 days.

### Sample size and data collection

The head of the household was interviewed for socio-demographic characteristics, assets and consumption expenditure. Information on education, occupation and enrolment in health insurance schemes were obtained for all individuals in the households. The respondent was interviewed for any illness in last 15 days or a hospitalization during last 365 days for any member of the household, along with reason for illness or hospitalization. An Out-patient (OP) care was considered to be consultation at any health facility for treatment of illness, whereas an admission at hospital was considered as hospitalization or in-patient (IP) care if there is atleast one night stay at health facility for treatment of illness. Reasons for illness was captured using symptoms reported by the respondent and diagnosis reported by the health care provider, in cases where care was sought. Information on treatment sought, its nature, OOP expenditure incurred and coping mechanisms were elicited. Care sought at multiple providers was captured. In order to ensure quality, supervisors revisited 5% of sampled households to check accuracy of data. Any discordance was resolved through discussion and re-visiting the household.

### Data analysis

The analysis focuses on assessing the effect of HI on self-reported illness events, extent and pattern of utilization, OOP burden due to healthcare payments and determinants of choice of healthcare providers.

Firstly, we analysed the data to assess the differences (in percent) in socio-demographic characteristics of households {i.e. state, locality of residence, religion of family, caste of family, Below Poverty Line (BPL) card status and wealth status} and individuals’ characteristics (i.e. age and gender) of the insured and uninsured sample groups. There are generally three approaches used to construct economic quintiles of population i.e. wealth based, income based and consumption expenditure based. We particularly used the 3^rd^ approach based on consumption expenditures. This approach is preferred over the income based approach because it is more stable in long term compared to income. Also, it requires a less number of items for data collection unlike to income based or wealth based approach [31]. Consumption expenditure was adjusted for household size and composition to compute monthly per capita consumption expenditure (MPCE). The latter was used to assess wealth status. To account for the regional variations within the survey sample, wealth quintiles were created separately for each state, and for rural and urban areas respectively.

Secondly, we assessed illness rate and hospitalization rate during the reference period of 15 days and 1 year respectively, by the insurance status and type of insurance scheme of the individuals. Thirdly, we assessed patterns of utilization of outpatient (OP) consultations and in-patient (IP) admissions across the different healthcare service providers (i.e. public, private and charitable), among those insured and not insured. In order to assess the effect of the insurance, we considered assessing the OP utilization patterns along with IP as some of Government schemes and SHI provide both OP and IP care coverage whereas RSBY and private insurance schemes limit the cover to IP care. Pairwise comparisons using Bonferroni adjustments were done to assess the statistical significant differences in the illness rates, hospitalization rates and choice of healthcare provider among the population enrolled under various insurance schemes.

#### Out-of-pocket healthcare payments and financial risk protection

We computed mean OOP expenditure for OP and IP by the insurance status of the individuals, type of insurance scheme and type of health facility utilized. T-test and Analysis of Variance (ANOVA) test were used to assess the statistical significance of differences observed.

We ascertained prevalence of catastrophic health expenditures (CHE) due to OOP payments for treatment both for OP consultations and hospitalizations, using a threshold of OOP expenditure in excess of 40% of household’s non-food consumption expenditure. Pairwise comparisons using Bonferroni adjustments were done to assess the statistical significant differences in the catastrophic health expenditures among the population enrolled under various insurance schemes.

#### Multivariate analysis

Multivariate regression with binary logit was used to ascertain the association of HI status of individuals as explanatory factor with outcomes like choice of care provider (i.e. public or non-public) and financial risk protection. We performed multivariate analysis to study the association between type of HI scheme as explanatory factor and choice of care provider as outcome. We also regressed RSBY enrolled and poorest 2 quintiles of uninsured households, considering the latter as proxy controls for RSBY population, with choice of care provider (public and private), hospitalization rates and prevalence of CHE. All the models built as a part of multivariate analysis were adjusted for potential covariates like caste, religion, number of household members, gender of patient, age of patient, education status of patient, type of illness, state and wealth quintile, to remove the confounding effect. Correlation between the covariates was assessed prior including them into the multivariate regression to avoid multi-collinearity. We report odds ratios (OR) as measure of association along with their 95% confidence limits and p-values.

### Ethical clearance

Ethical clearance for the study was obtained from Institute Ethics Committee of Post-Graduate Institute of Medical Education & Research, Chandigarh, India. Administrative approval was sought from administrative heads of health departments in all the concerned states. Written informed consent for participation was obtained from respondents in household survey.

## Results

### Sample characteristics

We collected data from randomly selected 12134 households and 62335 individuals in 3 states. Data on HI status was missing for 77 individuals, hence data was analysed for 62258 individuals. Socio-demographic characteristics of those insured and non-insured are given in Table 1. Overall, coverage of HI was found to be 20%, with highest in Gujarat (25%), followed by Haryana (23%) and UP (10%) respectively. Coverage of HI was also found to be higher for urban residents (25%), those belonging to age above 60 years (27%), Christian households (25%), and BPL families (37%) (Table 1).

**Table 1:** Socio-demographic characteristics of sample population by their health insurance status.

Characteristics		Insured		Not Insured		Total	
		n	%	n	%	n	%
Total		12745	20	49513	80	62258	100
**State**	Haryana	5147	23	17125	77	22272	100
	Gujarat	5958	25	17501	75	23459	100
	Uttar Pradesh	1640	10	14887	90	16527	100
**Area**	Rural	6957	18	31850	82	38807	100
	Urban	5680	25	17186	75	22866	100
	Slum	108	18	477	82	585	100
**Gender**	Male	6754	20	26217	80	32971	100
	Female	5991	20	23296	80	29287	100
**Age**	Below 5 years	938	15	5157	85	6095	100
	5–15 years	2163	19	9519	81	11682	100
	16–60 years	8613	21	32008	79	40621	100
	More than 60 years	1031	27	2829	73	3860	100
**Religion**	Hindu	12071	21	44562	79	56633	100
	Muslim	521	11	4324	89	4845	100
	Christian	46	25	136	75	182	100
	Sikh	95	18	435	82	530	100
	Other (specify)	12	18	56	82	68	100
**Caste**	Scheduled caste	2301	24	7390	76	9691	100
	Scheduled Tribe	2708	31	6140	69	8848	100
	OBC	3256	14	20463	86	23719	100
	General	4450	22	15347	78	19797	100
	Refuses to answer	30	15	173	85	203	100
**BPL Card**	Yes	5337	37	8896	63	14233	100
	No	7408	15	40617	85	48025	100
**Wealth Quintile**	1	2314	19	10117	81	12431	100
	2	2329	19	10128	81	12457	100
	3	2566	21	9896	79	12462	100
	4	2473	20	9992	80	12465	100
	5	3063	25	9380	75	12443	100

**Note:** OBC = other backward class

### Illness and hospitalization rates

Overall, we found significant differences in self-reported illness rates across the insured (13%) and uninsured (12%) population. Also, significant variations were observed in illness rates among those with different insurance coverage (p = <0.001). Illness rates were reported to be higher among the individuals enrolled under public HI schemes i.e. State Govt. employee schemes (21%), SHI scheme (14%) and RSBY (11%) in comparison to the population enrolled under private HI (9%) (Table 2). Pairwise comparisons were done using Bonferroni adjustments which suggest that differences in illness rates are statistically significant for all insurance schemes except between private mediclaim insurance and RSBY (Table 1A & B in S1 File).

**Table 2:** Illness in last 15 days and hospitalization status in last 1 year by health insurance status and type of health insurance scheme.

**Insurance Status**	**Illness Status (N = 62232)**		**p** **(chi-square)**	**Hospitalization (N = 62254)**		**p** **(chi-square)**
	**n**	**%**	0.007	**n**	**%**	0.006
Yes	1622	13		598	5	
No	5870	12		2053	4	
Total	7492	12		2651	4	
**Insurance Scheme**	**N = 12528**			**N = 12532**		
RSBY	677	11	<0.001	228	4	<0.001
Social Health Insurance	388	14		162	6	
State Govt. Schemes	388	21		95	5	
Private Insurance	138	9		97	7	
Total	1591	13		582	5	

**Note:** ‘N’ may not be same for different calculations within the table due to some missing information.

Similarly, there was a significant difference in hospitalization rate according to whether or not covered under HI (p = 0.007) and type of HI scheme (p = <0.001). Hospitalization rates in insured and uninsured population were 5% and 4% respectively. In contrast to illness rates which were higher among the people enrolled under public HI and lower among the private HI, hospitalization rates were higher among the individuals enrolled under private HI and lowest among individuals enrolled under public HI scheme RSBY (Table 2). Pairwise comparisons suggest that differences in hospitalization rates are statistically significant for RSBY scheme compared with Social Health Insurance and Private Mediclaim insurance. Whereas, the pairwise differences among State Government schemes, Social Health insurance and Private Mediclaim insurance are statistically insignificant after adjustment (Table 2A & B in S1 File).

### Service utilization

We observed that higher overall private sector utilization for OP care among the insured population, though, variations in utilization of health facilities among various HI scheme was observed. Public sector facilities were utilized at a higher rate by individuals enrolled under HI schemes of State Government (38%) followed by population enrolled under SHI and RSBY i.e. 24% and 17% respectively. Utilization of public sector facilities was minimum (6%) among population enrolled under private HI (Table 3). Findings of pairwise comparisons show public sector utilization for out-patient care are significantly different among the population enrolled under State Government schemes compared with RSBY and Private Mediclaim insurance and; Social Health insurance and Private Medicalim insurance (Table 3A & B in S1 File).

**Table 3:** Utilization by type of health care provider and health insurance scheme for illness in last 15 days and hospitalization in last 1 year.

Insurance Scheme	Type of Care Provider					
	Govt. Hospital/Clinic/Dispensary		Private Hospital/Clinic/Dispensary/Pharmacies		Total	
	n	%	n	%	n	%
**Illness last 15 days**						
RSBY	113	17	557	83	670	100
Social Health Insurance	43	24	138	76	181	100
State Govt. Schemes	30	38	49	62	79	100
Private Insurance	4	7	52	93	56	100
Total	190	19	796	81	986	100
**Hospitalization last 1 year**						
RSBY	64	25	188	75	252	100
Social Health Insurance	51	32	111	69	162	100
State Govt. Schemes	31	44	39	56	70	100
Private Insurance	7	8	77	92	84	100
Total	153	27	415	73	568	100

Similarly, private sector was predominantly used for hospitalization, by those enrolled under any HI scheme (i.e. public or private). Utilization of public sector hospitals for IP care was highest (44%) for population enrolled under schemes run by state Government followed by 31% in SHI and 25% in RSBY. Minimal utilization of public hospitals was seen for in-patient treatment among population enrolled under private HI (Table 3). Pairwise comparisons show public sector utilization for hospitalization are significantly different among the population enrolled under State Government schemes compared with RSBY and Private Mediclaim insurance and; Social Health insurance and Private Medicalim insurance (Table 4A & B in S1 File).

### Out-of-pocket payments

Mean OOP expenditures for OP care among insured and uninsured population was INR 961 (USD 16) and INR 840 (USD 14) respectively. OOP expenditures were higher among those enrolled under RSBY i.e. INR 1035 (USD 17) followed by SHI (INR 1027 (USD 17)), private HI (INR 717 (USD 12)) and least for state Government schemes (INR 405 (USD 7)). This could be seen in view of the fact that schemes like RSBY does not provide cover for OP treatment (Table 4). Though, these differences were not statistically significant tested using t-test and ANOVA methods.

**Table 4:** Out-of-pocket expenditures (in INR#) for healthcare payments and catastrophic health expenditures by insurance status and by type of health insurance scheme for illness in last 15 days and hospitalization in last 1 year.

Insurance Status	Out-of-pocket expenditures		Catastrophic Health Expenditure	
	Mean (INR#)	SE*	N	%
***Illness last 15 days***				
Insured	961	88	4	0.52
Non-Insured	840	39	21	0.50
Total	858	36	25	0.51
*Insurance Scheme*				
RSBY	1035	92	4	0.73
Social Health Insurance	1027	355	0	0
State Govt. Schemes	405	71	0	0
Private Insurance	717	112	0	0
Total	968	89	4	0.53
***Hospitalizations last 1 year***				
Insured	32573	5516	146	28
Not Insured	24788	1325	470	26
Total	26417	1560	616	26
*Insurance Scheme*				
RSBY	15687	1345	90	39
Social Health Insurance	30272	6144	22	16
State Govt. Schemes	47150	15829	13	21
Private Insurance	73508	31484	18	23
Total	32697	5640	143	28

*SE = Standard error

# = Indian National Rupee

Mean OOP expenditures for IP care among insured and uninsured population was INR 32,573 (USD 543) and INR 24,788 (USD 413), respectively. Using t-test, we found, these differences were not statistically significant (p = 0.17). Mean OOP expenditures for hospitalizations were highest for population enrolled under private insurance i.e. INR 73,508 (USD 1225) and lowest for population enrolled under RSBY i.e. INR 15,687 (USD 261) (Table 4). The differences in OOP expenditures between RSBY and Private insurance enrolees were statistically significant (p = 0.003)

### Catastrophic health expenditures

Among those who sought outpatient care, 1% had CHE irrespective of whether they are covered or not under any HI scheme (Table 4). In the event of hospitalization, prevalence of CHE was 28% and 26% among the insured and uninsured population respectively. Significant variation (p = <0.001) was observed for CHE by the type of HI scheme. Higher prevalence rate of CHE was observed for those enrolled under RSBY i.e. 39%, followed by private HI (23%) and state Government schemes (21%) respectively. Prevalence of CHE was least (16%) among the population enrolled under SHI schemes (Table 4).

### Multivariate results

We found that odds of utilization of non-public health facilities for OP consultation was insignificantly different (i.e. OR = 1.4, p = 0.130) among the insured and non-insured population (Table 5, Model 1; Model 1 in S1 File). Utilization of public health facilities for OP consultation was same for population enrolled under RSBY scheme and uninsured population belonged to lowest 2 SES quintiles (Table 5, Model 2; Model 2 in S1 File).

**Table 5:** Association of health insurance and type of health insurance with choice of care provider and financial risk protection (hospitalization).

Multivariate Regression (Binary Logit)	Insurance Status	OR	95% C.I. for OR		p	Model Description
			Lower	Upper		
Model 1	Insured	1.498	0.888	2.526	0.130	*Dependent Variable*:- **Care Provider for out-patient care** (Govt. Facility = 0, Non-Govt. = 1), *Explanatory Variable*:- Insurance Status (Insured = 1, Non-Insured = 0), *Adjusted for*: Caste, Religion, Number of household members, gender of patient, age of patient, education status of patient, type of illness, state and wealth quintile.
	Non-Insured	Reference				
Model 2	RSBY	1.16	0.56	2.42	0.689	*Dependent Variable*:- **Care Provider for out-patient care** (Govt. Facility = 1, Non-Govt. = 0), *Explanatory Variabl*e:- Insurance Scheme (RSBY = 1, Non-Insured population in quintile 1&2 = 0), *Adjusted fo*r: Caste, Religion, Number of household members, gender of patient, age of patient, education status of patient and type of illness.
	Quintile 1&2	Reference				
Model 3	Quintile 1&2	1.36	0.9	2.04	0.145	*Dependent Variable*:- **Hospitalization** (Hospitalized for illness = 1, Not hospitalized for illness = 0), *Explanatory Variable*:- Insurance Status (RSBY = 0, Non-Insured population in quintile 1&2 = 1), *Adjusted for*: Caste, Religion, Number of household members, gender of patient, age of patient, education status of patient, type of illness and state.
	RSBY	Reference				
Model 4	Non-Insured	1.06	0.81	1.38	0.683	*Dependent Variable*:- **Care Provider for in-patient care** (Govt. Facility = 1, Non-Govt. = 0), *Explanatory Variable*:- Insurance Status (Insured = 1, Non-Insured = 0), *Adjusted for*: Caste, Religion, Number of household members, gender of patient, age of patient, education status of patient, type of illness, state and wealth quintile.
	Insured	Reference				
Model 5	Quintile 1&2	1.72	0.70	4.19	0.235	*Dependent Variable*:- **Care Provider for in-patient care** (Govt. Facility = 1, Non-Govt. = 0), *Explanatory Variable*:- Insurance Scheme (RSBY = 0, Non-Insured population in quintile 1&2 = 1), *Adjusted for*: Caste, Religion, Number of household members, gender of patient, age of patient, education status of patient, type of illness and state.
	RSBY	Reference				
Model 6	Non-Insured	1.03	0.78	1.36	0.844	*Dependent Variable*:- **Catastrophic health expenditure due to hospitalization** (Yes = 1, No = 0), *Explanatory Variable*:- Insurance status (Insured = 1, Non-Insured = 0), *Adjusted for*: Caste, Religion, Number of household members, gender of patient, age of patient, education status of patient, type of health facility, hospitalization days, type of illness, state and wealth quintile.
	Insured	Reference				
Model 7	RSBY	2.47	1.15	5.29	0.021	*Dependent Variable*:- **Catastrophic health expenditure due to hospitalization** (Yes = 1, No = 0), *Explanatory Variable*:- Insurance Scheme (RSBY = 1, Non-RSBY = 0), *Adjusted for*: Caste, Religion, Number of household members, gender of patient, age of patient, education status of patient, type of health facility, hospitalization days, type of illness and state.
	Other Insurances	Reference				
Model 8	RSBY	2.74	0.75	9.96	0.126	*Dependent Variable*:- **Catastrophic health expenditure due to hospitalization** (Yes = 1, No = 0), *Explanatory Variable*:- Insurance Scheme (RSBY = 1, Non-Insured population in quintile 1&2 = 1), *Adjusted for*: Caste, Religion, Number of household members, gender of patient, age of patient, education status of patient, type of health facility, hospitalization days, type of illness and state.
	Quintile 1&2	Reference				

**Note:** In Model 1 and 2 outcome variable was choice of healthcare provider with key explanatory variable as insurance status for model 1 and population enrolled under RSBY vs uninsured population in wealth quintile 1 and 2 for model 2. In model 3, outcome variable was hospitalization for illness and explanatory variable was population enrolled under RSBY vs non-insured population in wealth quintile 1 and 2. Model 4 and 5 were fitted using outcome variable as choice of healthcare provider for in-patient care and explanatory variable as insurance status and; population enrolled under RSBY vs uninsured population in wealth quintile 1 and 2, respectively. Model 6, 7 and 8 were fitted using outcome variable as catastrophic health expenditures faced in case of hospitalization events. In model 6, explanatory variables used was insurance status (i.e. insured vs uninsured population). In model 7, explanatory variable was population enrolled under RSBY vs other insurances. In model 8, explanatory variable was population enrolled under RSBY vs non-insured population in wealth quintile 1 and 2.

We observed that the rate of hospitalization between the population enrolled under RSBY scheme and population belonged to lowest 2 SES quintiles was statistically insignificant (Table 5, Model 3; Model 3 in S1 File). No significant association was found between choice of public and private care providers for hospitalization and insurance status and; and among RSBY compared to non-insured population belonged to lowest 2 SES quintiles (Table 5, Model 4&5; Model 4&5 in S1 File). CHEs for hospitalization were insignificantly associated with insurance status of individuals but significantly associated with type of HI schemes. Population enrolled under RSBY had a significantly higher odds (OR = 2.47; 95% CI: 1.15, 5.29) for having CHE compared to population enrolled under other HIs (Table 5, Model 7; Model 7 in S1 File).

## Discussion

Moving over the rhetoric for the need for UHC, several policy discourses in India currently focus on ‘how’ to achieve UHC [4]. The question of whether to go via the supply-side funded public sector route or using demand side financing mechanisms such as recently introduced publicly financed health insurance schemes becomes inevitable. In order to answer the latter, it is imperative to evaluate the existing schemes in terms of their impact on increasing access to healthcare utilization and providing financial risk protection to targeted groups. The extent of evidence so far, especially for financial risk protection, is inadequate. Moreover, the direction of findings is ambiguous.

In this study, we report analysis from 12134 households and 62335 individuals in Haryana, Gujarat and UP state. We found a statistically significant difference among the insured and non-insured; as well as among the RSBY enrolled and non-RSBY enrolled bottom 2 poorest quintiles–in terms of either rates of reporting illness, hospitalization or the extent to which public sector was used for hospitalization. Surprisingly, those enrolled under RSBY had a significantly higher odds of facing catastrophic health expenditures.

### Strengths

Firstly, choice of our states increases the external validity of our findings. One state was chosen each from 3 brackets of per capita income, where Haryana represented the top-most bracket, Gujarat though an advanced state represented middle per capita income bracket and UP represented the bottom-most bracket, which is an EAG state too. Secondly, we had a reasonably large sample size of 12134 households and 62335 individuals to undertake our statistical analysis. Thirdly, our study is not merely limited to evaluate the effect of some specific HI scheme rather we assessed the variations in healthcare utilization for any illness, utilization of public health facilities, financial risk protection etc. in the presence and absence of HI. Moreover, we also assessed variations in the above mentioned indicators comparing publicly financed schemes like RSBY with SHI, Government HI schemes and private HI.

### Comparison with existing evidence

We found the coverage of HI schemes at aggregate level to be 20%. Haryana and Gujarat had almost similar levels of coverage with almost one-fourth population covered under any form of HI but UP had only one-tenth population covered under HI. Our pooled estimates are close to estimates given by previous studies done in India. A study utilizing the administrative data of schemes (2012) and the annual report (2013–14) published by Insurance Regulatory and Development Authority (IRDA) of India estimated the coverage of HI in India to be 25% and 18% (216 million), respectively [10, 32]. Findings of 71^st^ round of NSS also depicted that 14% and 18% of rural and urban population, respectively, had coverage of health expenditure support [33]. A study assessing the HI models in India also reported the coverage of HI in 2010 to be 25% [5]. Previous studies done for north Indian state reported higher levels of OOP expenditures and hence, more likelihood of CHE among the insured population [34, 35]. Our study findings also depict the same particularly for population enrolled under RSBY scheme.

We collected the data for this study from April to September, 2014, which overlaps with the reference period of 71^st^ round (Morbidity survey) of NSS i.e. January to June, 2014 and therefore, our estimates can be compared with estimates of NSS 71^st^ round [33]. We estimated the illness rate and hospitalization rates to be 12% and 4% at pooled level. Pooled findings for the states Haryana, Gujarat and UP from NSS 71^st^ round show that illness rate and hospitalization rate are 8% and 4% respectively. While the estimate of hospitalization rate is exactly same as given by NSS, a 4 point difference in illness rate is observed between NSS estimates and our findings. A potential reason for NSS estimate to be on the lower side could be the methodological changes done by NSS in 71^st^ round compared to previous surveys. In 71^st^ round, only illnesses/disabilities whose onset was within the reference period i.e. in the last 15 days of survey were captured. Pre-existing illnesses were considered as chronic and left subject to person with illness is on treatment for more than 1 month [33]. We also observed variations in illness and hospitalization rates for the population enrolled under different HI schemes. Population enrolled under SHI and Government schemes reported higher illness rates of 14% and 21%, respectively, relative to RSBY (11%) and private insurances (9%). Low illness rate among population insured under private insurance could be explained with higher rate of hospitalization i.e. 7% among the same population. Moreover, the cashless nature of private insurance might have some effect on switching the patients from OP setting (which generally is not covered in private insurances) to hospitalization. Low illness rates (in last 15 days) among population insured under RSBY could be related with fact that OP treatment is not covered in RSBY till date which results in financial hardship hampering the treatment seeking particularly in case of population belonging to poorest quintiles.

Our study showed, population enrolled under Government schemes and SHI utilized the public hospitals/clinics both for OP and IP care compared to population enrolled under RSBY and private insurances. This probably is a reflection of better gatekeeping mechanisms in SHI and Government schemes. A critical assessment of HI models in India discussed the usefulness of gatekeeping mechanisms of referral systems in SHI in controlling cost of care [5]. We found more odds of utilizing public health facilities for OP treatment by uninsured population which were statistically significant also but insignificant differences for choice of care provider for hospitalization were observed. One previous study done in two south Indian states revealed utilization of private health facilities increased in one of the state in post-insurance period [36].

Several studies were done in India which measured the impact of insurance but they targeted the some specific state insurance schemes [12, 21, 22, 36, 37]. These studies employed a pre-post design to see the impact mainly relying on the data covered under different NSS round, though some studies did primary data collection for the post-insurance period [36, 37]. We could not find any study estimating the CHE in a cross-sectional design like ours. After controlling for potential confounders, multivariate results from our study depicts insignificant difference in the prevalence of CHE between the insured and uninsured population (Table 5, Model 6) whereas we found significant high odds (OR = 2.47, p = 0.021) of CHE among the population enrolled under RSBY compared to other insurance schemes (Table 5, Model 7). This could be viewed in the light that despite that the population enrolled under RSBY gets insurance coverage in crude sense but with a limit of INR 30,000 which makes it inefficient in terms of providing financial risk protection to the targeted population. We also regressed RSBY population with poorest 2 quintiles of uninsured population considering it as proxy control. Insignificant differences in odds of CHE were observed between these (Table 5, Model 8).

Health insurance schemes in India can be broadly classified into 3 categories i.e. tax-funded RSBY scheme; mandatory social health insurance (SHI) Scheme and Government Insurance schemes; and voluntary private health insurance (VHI) schemes. Currently, majority of the population which is covered under any form of insurance includes RSBY (i.e. 77%), whereas SHI and VHI constitute a share of 16% and 7%, respectively [38]. In terms of the incentives for self-selection, Government schemes and SHI are mandatory in nature i.e. one gets enrolled as a benefit of their employment, so there is no chance of self-selection. In contrast, the enrolment under private insurance scheme is completely voluntary in nature, and hence there is a risk of self-selection. However, its overall proportion in the share of those insured in India, as well as in our study sample (less than 10%). Hence, it is unlikely to cause a significant effect on overall healthcare utilization even if adverse selection is present. RSBY scheme extends its benefits by default for the BPL population but one (or household) has to enroll himself every year by applying for a smart card. Enrolment camps are organized every year at village level within the district(s), generally at a public place. While there is no premium attached to enrolment, individuals/households get excluded out of scheme if they do not mark presence on the specific days of enrolment camps. However, existing evidence suggest absence of any large scale adverse selection in RSBY [39–41]. A recent systematic review of existing impact evaluations of publicly funded health insurance schemes also suggests absence of any significant self-selection [20].

Despite such arguments effect of self-selection bias in results cannot be ruled out. We acknowledge that adopting a more robust research design like Randomized Controlled Trial (RCT) or quasi-experimental would have been a better choice to assess the causality but use of cross-sectional data after adjusting for potential confounders give us some degree of confidence to assess of role of insurance in healthcare utilization and providing financial risk protection, if not impact. In order to make those insured under RSBY and those not-insured, we compared the RSBY enrolled households with bottom 40% of the controls. The latter choice is influenced by the fact that RSBY covers the below poverty line population which comprises about bottom one-third of total Indian population. For example, model 3 in Table 5 specifically depict that there is no difference in hospitalization rates among population enrolled under RSBY against the uninsured population with similar wealth status (wealth quintile 1 and 2). With no difference in healthcare utilization among the RSBY enrolees and non-enrolees in our results, it is unlikely to have a possibility of adverse selection. Finally, in our analysis, we also control for the potential factors which are stated to be associated with self-selection [42–44]. These factors include socio-economic factors like caste and religion; individual characteristics like gender of patient, age of patient and education status of patient; household characteristics like number of household members and wealth status; other factors like type of illness, type of health facility, hospitalization days and state.

Our study findings also hold significant importance for future research which may be done in the context of Government of India’s Ayushman Bharat Prime Minister’s Jan Aarogya Yojana [45]. The scheme aims to cover bottom 40% of the population, based on a set of means-testing criteria, with an insurance coverage for hospitalization. As the scheme is still in its early implementation phase, there is a need to re-engineer the data systems such that such indications of self-selection are derived from routine enrolment data. Further, researchers involved in doing interim and end-term impact evaluations should consider introducing designs such that more robust control population is selected so that causal implications are more robust.

### Limitations

Randomized controlled trials (RCTs) are considered as best research designs to measure the impact as they control for both observed and unobserved factors affecting outcome. In the absence of RCTs, even pre-post with a control group or quasi-experimental are resorted to, in order to assess the impact. One of the limitation of our study is that our research design is cross-sectional in nature which restricts us to establish the causality in robust manner and evaluate the impact of HI or particularly publicly financed schemes over the period of time. This is related to the fact that the intervention was implemented in all areas, rather than any randomized allocation of treatment. Other sources of data could have been used to generate information of the pre-intervention period [46]. However, inherent differences in survey methodologies rule out the former possibility. Hence, we used the cross-sectional data and tried to adjust for known confounders in the analysis. We presume that some health system factors also influence the healthcare utilization and OOP healthcare payments, be it induced demand for healthcare mainly in private sector or unavailability of some procedures/specialization specifically in public sector, which needs to be adjusted to assess the impact in true terms. We did not adjust for such factors in our study which could be seen as another limitation. Also, based on results of our study, we could not comment neither on presence and extent of supplier’s induced demand. We recommend more focussed research for overcoming the limitations in our study.

### Policy implications

Our study findings in terms of no relationship between enrolment in a publicly financed health insurance scheme and utilization of care or financial risk protection poses significant question to the policy impetus being provided towards expanding the coverage of these demand-side funding mechanisms. Other studies also offer similar conclusions [20]. Moreover, data from various rounds of the NSS surveys also indicate towards rising trends of OOP expenditure. On the contrary, focal interventions where public health care delivery system was augmented have shown encouraging results. One glaring example is the promotion of institutional deliveries as part of the National Health Mission interventions [47, 48]. Moreover, public sector delivery has been shown to be efficient [49]. A combination of these supply and demand-side strengthening mechanisms have resulted in a dramatic increase in institutional deliveries, especially contributed through public sector, coupled with a reduction in OOP expenditures for maternity care. This points towards positive effects of public sector supply side strengthening. This implies that public sector has a capacity to universalize provision of services, given the requisite financial allocations.

Secondly, the findings could also be a result of the design of the insurance benefit packages. While evidence shows that majority of the OOP expenditures is on account of outpatient consultation, the same is not covered under current publicly financed HI schemes. Secondly, the primary care is not covered in existing schemes, which focus primarily on tertiary and secondary care. The existing policy needs to realign to these realities. There is a need to focus on strengthening provision of primary care.

## Supporting information

S1 File Supporting information. (DOCX)
