# Supplementary material for: Role of insurance in determining utilization of healthcare and financial risk protection in India
Source: PLoS One. 2019 Feb 5;14(2):e0211793. doi: 10.1371/journal.pone.0211793 (PMC6363222; doi:10.1371/journal.pone.0211793)
Supplement: S1 File — (DOCX) [file pone.0211793.s001.docx]

**Results from Pairwise Comparisons using Bonferroni adjustments**

**Table 1A: Pairwise Comparisons for Illness Rates by Insurance Scheme**

| **Estimates** | | | | |
| --- | --- | --- | --- | --- |
| Insurance Type | Illness rate | Std. Error | 95% Wald Confidence Interval | |
|  |  |  | Lower | Upper |
| RSBY | .11 | .004 | .10 | .11 |
| Social Health Insurance | .14 | .006 | .12 | .15 |
| State Govt. Schemes | .21 | .009 | .19 | .23 |
| Private Insurance (Mediclaim) | .09 | .008 | .08 | .11 |

**Table 1B: Pairwise Comparisons for Illness Rates by Insurance Scheme**

| **Pairwise Comparisons** | | | | | | | |
| --- | --- | --- | --- | --- | --- | --- | --- |
| (I) Insurance Type | (J) Insurance Type | Mean Difference (I-J) | Std. Error | df | Bonferroni Sig. | 95% Wald Confidence Interval for Difference | |
|  |  |  |  |  |  | Lower | Upper |
| RSBY | Social Health Insurance | -.03^a^ | .008 | 1 | .000 | -.05 | -.01 |
|  | State Govt. Schemes | -.10^a^ | .010 | 1 | .000 | -.13 | -.07 |
|  | Private Insurance (Mediclaim) | .01 | .009 | 1 | .794 | -.01 | .04 |
| Social Health Insurance | RSBY | .03^a^ | .008 | 1 | .000 | .01 | .05 |
|  | State Govt. Schemes | -.07^a^ | .011 | 1 | .000 | -.10 | -.04 |
|  | Private Insurance (Mediclaim) | .04^a^ | .010 | 1 | .000 | .02 | .07 |
| State Govt. Schemes | RSBY | .10^a^ | .010 | 1 | .000 | .07 | .13 |
|  | Social Health Insurance | .07^a^ | .011 | 1 | .000 | .04 | .10 |
|  | Private Insurance (Mediclaim) | .11^a^ | .012 | 1 | .000 | .08 | .14 |
| Private Insurance (Mediclaim) | RSBY | -.01 | .009 | 1 | .794 | -.04 | .01 |
|  | Social Health Insurance | -.04^a^ | .010 | 1 | .000 | -.07 | -.02 |
|  | State Govt. Schemes | -.11^a^ | .012 | 1 | .000 | -.14 | -.08 |
| Pairwise comparisons of estimated marginal means based on the original scale of dependent variable Ill, in last two weeks? | | | | | | | |
| a. The mean difference is significant at the .05 level. | | | | | | | |

**Table 2A: Pairwise Comparisons for Hospitalization Rates by Insurance Scheme**

| **Estimates** | | | | |
| --- | --- | --- | --- | --- |
| Insurance Type | Hospitalization rate | Std. Error | 95% Wald Confidence Interval | |
|  |  |  | Lower | Upper |
| RSBY | .04 | .002 | .03 | .04 |
| Social Health Insurance | .06 | .005 | .05 | .07 |
| State Govt. Schemes | .05 | .006 | .04 | .07 |
| Private Insurance (Mediclaim) | .07 | .007 | .06 | .09 |

**Table 2B: Pairwise Comparisons for Hospitalization Rates by Insurance Scheme**

| **Pairwise Comparisons** | | | | | | | |
| --- | --- | --- | --- | --- | --- | --- | --- |
| (I) Insurance Type | (J) Insurance Type | Mean Difference (I-J) | Std. Error | df | Bonferroni Sig. | 95% Wald Confidence Interval for Difference | |
|  |  |  |  |  |  | Lower | Upper |
| RSBY | Social Health Insurance | -.02^a^ | .005 | 1 | .000 | -.04 | -.01 |
|  | State Govt. Schemes | -.01 | .007 | 1 | .186 | -.03 | .00 |
|  | Private Insurance (Mediclaim) | -.03^a^ | .008 | 1 | .000 | -.05 | -.01 |
| Social Health Insurance | RSBY | .02^a^ | .005 | 1 | .000 | .01 | .04 |
|  | State Govt. Schemes | .01 | .008 | 1 | 1.000 | -.01 | .03 |
|  | Private Insurance (Mediclaim) | -.01 | .009 | 1 | 1.000 | -.03 | .01 |
| State Govt. Schemes | RSBY | .01 | .007 | 1 | .186 | .00 | .03 |
|  | Social Health Insurance | -.01 | .008 | 1 | 1.000 | -.03 | .01 |
|  | Private Insurance (Mediclaim) | -.02 | .010 | 1 | .383 | -.04 | .01 |
| Private Insurance (Mediclaim) | RSBY | .03^a^ | .008 | 1 | .000 | .01 | .05 |
|  | Social Health Insurance | .01 | .009 | 1 | 1.000 | -.01 | .03 |
|  | State Govt. Schemes | .02 | .010 | 1 | .383 | -.01 | .04 |
| Pairwise comparisons of estimated marginal means based on the original scale of dependent variable Hospitalized_1year | | | | | | | |
| a. The mean difference is significant at the .05 level. | | | | | | | |

**Table 3A: Pairwise Comparisons for Utilization of Healthcare Provider for Out-patient Care by Insurance Scheme**

| **Estimates** | | | | |
| --- | --- | --- | --- | --- |
| Insurance Type | Public Sector Utilization | Std. Error | 95% Wald Confidence Interval | |
|  |  |  | Lower | Upper |
| RSBY | .17 | .014 | .14 | .20 |
| Social Health Insurance | .24 | .032 | .18 | .30 |
| State Govt. Schemes | .38 | .055 | .28 | .49 |
| Private Insurance (Mediclaim) | .07 | .034 | .03 | .17 |

**Table 3B: Pairwise Comparisons for Utilization of Healthcare Provider for Out-patient Care by Insurance Scheme**

| **Pairwise Comparisons** | | | | | | | |
| --- | --- | --- | --- | --- | --- | --- | --- |
| (I) Insurance Type | (J) Insurance Type | Mean Difference (I-J) | Std. Error | df | Bonferroni Sig. | 95% Wald Confidence Interval for Difference | |
|  |  |  |  |  |  | Lower | Upper |
| RSBY | Social Health Insurance | -.07 | .035 | 1 | .285 | -.16 | .02 |
|  | State Govt. Schemes | -.21^a^ | .056 | 1 | .001 | -.36 | -.06 |
|  | Private Insurance (Mediclaim) | .10 | .037 | 1 | .055 | .00 | .20 |
| Social Health Insurance | RSBY | .07 | .035 | 1 | .285 | -.02 | .16 |
|  | State Govt. Schemes | -.14 | .063 | 1 | .146 | -.31 | .02 |
|  | Private Insurance (Mediclaim) | .17^a^ | .047 | 1 | .002 | .04 | .29 |
| State Govt. Schemes | RSBY | .21^a^ | .056 | 1 | .001 | .06 | .36 |
|  | Social Health Insurance | .14 | .063 | 1 | .146 | -.02 | .31 |
|  | Private Insurance (Mediclaim) | .31^a^ | .065 | 1 | .000 | .14 | .48 |
| Private Insurance (Mediclaim) | RSBY | -.10 | .037 | 1 | .055 | -.20 | .00 |
|  | Social Health Insurance | -.17^a^ | .047 | 1 | .002 | -.29 | -.04 |
|  | State Govt. Schemes | -.31^a^ | .065 | 1 | .000 | -.48 | -.14 |
| Pairwise comparisons of estimated marginal means based on the original scale of dependent variable Provider1_Cat_New | | | | | | | |
| a. The mean difference is significant at the .05 level. | | | | | | | |

**Table 4A: Pairwise Comparisons for Utilization of Healthcare Provider for In-patient Care by Insurance Scheme**

| **Estimates** | | | | |
| --- | --- | --- | --- | --- |
| Insurance Type | Public Sector Utilization | Std. Error | 95% Wald Confidence Interval | |
|  |  |  | Lower | Upper |
| RSBY | .25 | .027 | .20 | .31 |
| Social Health Insurance | .31 | .036 | .25 | .39 |
| State Govt. Schemes | .44 | .059 | .33 | .56 |
| Private Insurance (Mediclaim) | .08 | .030 | .04 | .16 |

**Table 4B: Pairwise Comparisons for Utilization of Healthcare Provider for In-patient Care by Insurance Scheme**

| **Pairwise Comparisons** | | | | | | | |
| --- | --- | --- | --- | --- | --- | --- | --- |
| (I) Insurance Type | (J) Insurance Type | Mean Difference (I-J) | Std. Error | df | Bonferroni Sig. | 95% Wald Confidence Interval for Difference | |
|  |  |  |  |  |  | Lower | Upper |
| RSBY | Social Health Insurance | -.06 | .046 | 1 | 1.000 | -.18 | .06 |
|  | State Govt. Schemes | -.19^a^ | .065 | 1 | .023 | -.36 | -.02 |
|  | Private Insurance (Mediclaim) | .17^a^ | .041 | 1 | .000 | .06 | .28 |
| Social Health Insurance | RSBY | .06 | .046 | 1 | 1.000 | -.06 | .18 |
|  | State Govt. Schemes | -.13 | .070 | 1 | .397 | -.31 | .06 |
|  | Private Insurance (Mediclaim) | .23^a^ | .047 | 1 | .000 | .11 | .36 |
| State Govt. Schemes | RSBY | .19^a^ | .065 | 1 | .023 | .02 | .36 |
|  | Social Health Insurance | .13 | .070 | 1 | .397 | -.06 | .31 |
|  | Private Insurance (Mediclaim) | .36^a^ | .067 | 1 | .000 | .18 | .54 |
| Private Insurance (Mediclaim) | RSBY | -.17^a^ | .041 | 1 | .000 | -.28 | -.06 |
|  | Social Health Insurance | -.23^a^ | .047 | 1 | .000 | -.36 | -.11 |
|  | State Govt. Schemes | -.36^a^ | .067 | 1 | .000 | -.54 | -.18 |
| Pairwise comparisons of estimated marginal means based on the original scale of dependent variable Facility_Type_New | | | | | | | |
| a. The mean difference is significant at the .05 level. | | | | | | | |

**Full Models for Table 5 in manuscript**

**Model 1**

| Factors | B | S.E. | Wald | df | Sig. | Exp(B) | 95% C.I. for EXP(B) | |
| --- | --- | --- | --- | --- | --- | --- | --- | --- |
|  |  |  |  |  |  |  | Lower | Upper |
| Insured | .404 | .267 | 2.292 | 1 | .130 | 1.498 | .888 | 2.526 |
| Caste- General category |  |  | 1.199 | 3 | .753 |  |  |  |
| Caste- Schedule caste | -.091 | .257 | .126 | 1 | .723 | .913 | .552 | 1.510 |
| Caste- Schedule Tribe | .128 | .372 | .119 | 1 | .731 | 1.137 | .548 | 2.359 |
| Caste- Other Backward class | .149 | .240 | .385 | 1 | .535 | 1.161 | .725 | 1.860 |
| Religion- Others |  |  | 3.424 | 3 | .331 |  |  |  |
| Religion- Hindu | .965 | .616 | 2.449 | 1 | .118 | 2.624 | .784 | 8.786 |
| Religion- Muslim | 20.358 | 28413.320 | .000 | 1 | .999 | 694096141.794 | 0.000 |  |
| Religion- Christian | -1.032 | 1.056 | .956 | 1 | .328 | .356 | .045 | 2.822 |
| Household size- Less than 5 members | -.019 | .184 | .011 | 1 | .917 | .981 | .685 | 1.406 |
| Gender- Female | .143 | .185 | .596 | 1 | .440 | 1.153 | .803 | 1.657 |
| Education- Literate | .071 | .219 | .104 | 1 | .747 | 1.073 | .698 | 1.649 |
| Age- > 60 years |  |  | .929 | 3 | .818 |  |  |  |
| Age- Below 5 years | -19.635 | 11998.776 | .000 | 1 | .999 | .000 | 0.000 |  |
| Age- 5-15 years | -19.623 | 11998.776 | .000 | 1 | .999 | .000 | 0.000 |  |
| Age- 16-60 years | -19.872 | 11998.776 | .000 | 1 | .999 | .000 | 0.000 |  |
| Disease- Other diseases |  |  | 5.578 | 3 | .134 |  |  |  |
| Disease- Communicable and MCH related | -.144 | .209 | .474 | 1 | .491 | .866 | .575 | 1.304 |
| Disease- Non-communicable | -.512 | .224 | 5.236 | 1 | .022 | .599 | .387 | .929 |
| Disease- Injuries | .125 | .664 | .036 | 1 | .850 | 1.134 | .309 | 4.162 |
| State- Uttar Pradesh |  |  | 3.496 | 2 | .174 |  |  |  |
| State- Haryana | -.384 | .284 | 1.822 | 1 | .177 | .681 | .390 | 1.189 |
| State- Gujarat | -.469 | .265 | 3.137 | 1 | .077 | .626 | .373 | 1.051 |
| Wealth Quintile- Poorest |  |  | 9.292 | 4 | .054 |  |  |  |
| Wealth Quintile- Poor | -.379 | .311 | 1.488 | 1 | .223 | .684 | .372 | 1.259 |
| Wealth Quintile- Middle | -.157 | .302 | .270 | 1 | .603 | .855 | .473 | 1.545 |
| Wealth Quintile- Rich | -.181 | .309 | .344 | 1 | .557 | .834 | .455 | 1.528 |
| Wealth Quintile- Richest | .486 | .344 | 1.996 | 1 | .158 | 1.625 | .829 | 3.188 |
| Constant | 21.105 | 11998.776 | .000 | 1 | .999 | 1465193997.686 |  |  |

**Model 2**

| Factors | B | S.E. | Wald | df | Sig. | Exp(B) | 95% C.I. for EXP(B) | |
| --- | --- | --- | --- | --- | --- | --- | --- | --- |
|  |  |  |  |  |  |  | Lower | Upper |
| Insured with RSBY | -.150 | .374 | .160 | 1 | .689 | 1.16 | 0.56 | 2.42 |
| Caste- General category |  |  | .010 | 3 | 1.000 |  |  |  |
| Caste- Schedule caste | .006 | .325 | .000 | 1 | .986 | 1.006 | .532 | 1.900 |
| Caste- Schedule Tribe | .018 | .388 | .002 | 1 | .963 | 1.018 | .476 | 2.176 |
| Caste- Other Backward class | -.012 | .303 | .002 | 1 | .969 | .988 | .545 | 1.791 |
| Religion- Others |  |  | 1.394 | 3 | .707 |  |  |  |
| Religion- Hindu | -.740 | .627 | 1.394 | 1 | .238 | .477 | .140 | 1.629 |
| Religion- Muslim | -20.372 | 28410.058 | .000 | 1 | .999 | .000 | 0.000 |  |
| Religion- Christian | -20.185 | 40192.970 | .000 | 1 | 1.000 | .000 | 0.000 |  |
| Household size- Less than 5 members | .187 | .217 | .741 | 1 | .389 | 1.206 | .787 | 1.846 |
| Gender- Female | -.153 | .218 | .490 | 1 | .484 | .858 | .559 | 1.317 |
| Education- Literate | .072 | .255 | .079 | 1 | .779 | 1.074 | .652 | 1.771 |
| Age- > 60 years |  |  | .824 | 3 | .844 |  |  |  |
| Age- Below 5 years | 19.891 | 16195.882 | .000 | 1 | .999 | 435254353.786 | 0.000 |  |
| Age- 5-15 years | 19.825 | 16195.882 | .000 | 1 | .999 | 407362227.118 | 0.000 |  |
| Age- 16-60 years | 20.109 | 16195.882 | .000 | 1 | .999 | 540832911.564 | 0.000 |  |
| Disease- Other diseases |  |  | 6.081 | 3 | .108 |  |  |  |
| Disease- Communicable and MCH related | -.014 | .243 | .003 | 1 | .955 | .986 | .612 | 1.589 |
| Disease- Non-communicable | .613 | .271 | 5.124 | 1 | .024 | 1.846 | 1.086 | 3.140 |
| Disease- Injuries | .231 | .682 | .115 | 1 | .735 | 1.260 | .331 | 4.795 |
| Wealth Quintile- Poorest |  |  | 7.985 | 4 | .092 |  |  |  |
| Wealth Quintile- Poor | .507 | .413 | 1.510 | 1 | .219 | 1.660 | .740 | 3.727 |
| Wealth Quintile- Middle | .941 | .388 | 5.889 | 1 | .015 | 2.561 | 1.198 | 5.475 |
| Wealth Quintile- Rich | .283 | .389 | .530 | 1 | .467 | 1.328 | .619 | 2.847 |
| Wealth Quintile- Richest | .623 | .390 | 2.547 | 1 | .111 | 1.865 | .867 | 4.007 |
| Constant | -21.940 | 16195.882 | .000 | 1 | .999 | .000 |  |  |

**Model 3**

| Factors | B | S.E. | Wald | df | Sig. | Exp(B) | 95% C.I. for EXP(B) | |
| --- | --- | --- | --- | --- | --- | --- | --- | --- |
|  |  |  |  |  |  |  | Lower | Upper |
| Insured with RSBY | .305 | .209 | 2.124 | 1 | .145 | 1.357 | .900 | 2.045 |
| Caste- General category |  |  | 25.201 | 3 | .000 |  |  |  |
| Caste- Schedule caste | -.137 | .184 | .550 | 1 | .458 | .872 | .608 | 1.251 |
| Caste- Schedule Tribe | -.837 | .213 | 15.505 | 1 | .000 | .433 | .285 | .657 |
| Caste- Other Backward class | .094 | .163 | .330 | 1 | .566 | 1.098 | .798 | 1.512 |
| Religion- Others |  |  | 2.041 | 3 | .564 |  |  |  |
| Religion- Hindu | -.356 | .317 | 1.268 | 1 | .260 | .700 | .376 | 1.302 |
| Religion- Muslim | .940 | 1.083 | .753 | 1 | .386 | 2.559 | .306 | 21.369 |
| Religion- Christian | -18.301 | 9294.518 | .000 | 1 | .998 | .000 | 0.000 |  |
| Household size- Less than 5 members | .006 | .120 | .002 | 1 | .961 | 1.006 | .795 | 1.273 |
| Gender- Female | -.448 | .125 | 12.736 | 1 | .000 | .639 | .500 | .817 |
| Education- Literate | .013 | .147 | .008 | 1 | .931 | 1.013 | .759 | 1.351 |
| Age- > 60 years |  |  | 18.845 | 3 | .000 |  |  |  |
| Age- Below 5 years | -18.507 | 4064.484 | .000 | 1 | .996 | .000 | 0.000 |  |
| Age- 5-15 years | -1.088 | .277 | 15.379 | 1 | .000 | .337 | .196 | .580 |
| Age- 16-60 years | -.284 | .203 | 1.953 | 1 | .162 | .753 | .506 | 1.121 |
| State- Uttar Pradesh |  |  | 1.030 | 2 | .598 |  |  |  |
| State- Haryana | .168 | .209 | .650 | 1 | .420 | 1.183 | .786 | 1.782 |
| State- Gujarat | .157 | .167 | .886 | 1 | .347 | 1.171 | .843 | 1.625 |
| Constant | -2.425 | .269 | 81.517 | 1 | .000 | .088 |  |  |

**Model 4**

| Factors | B | S.E. | Wald | df | Sig. | Exp(B) | 95% C.I. for EXP(B) | |
| --- | --- | --- | --- | --- | --- | --- | --- | --- |
|  |  |  |  |  |  |  | Lower | Upper |
| Insured | .056 | .137 | .167 | 1 | .683 | 1.058 | .808 | 1.385 |
| Caste- General category |  |  | 1.773 | 3 | .621 |  |  |  |
| Caste- Schedule caste | .169 | .173 | .955 | 1 | .328 | 1.185 | .843 | 1.664 |
| Caste- Schedule Tribe | .286 | .262 | 1.194 | 1 | .275 | 1.331 | .797 | 2.224 |
| Caste- Other Backward class | .050 | .130 | .150 | 1 | .698 | 1.052 | .815 | 1.357 |
| Religion- Others |  |  | 7.202 | 4 | .126 |  |  |  |
| Religion- Hindu | -.414 | .226 | 3.346 | 1 | .067 | .661 | .424 | 1.030 |
| Religion- Muslim | 18.891 | 40192.969 | .000 | 1 | 1.000 | 160058049.868 | 0.000 |  |
| Religion- Christian | -.282 | .577 | .239 | 1 | .625 | .754 | .243 | 2.339 |
| Caste- General category | -2.506 | 1.285 | 3.803 | 1 | .051 | .082 | .007 | 1.013 |
| Household size- Less than 5 members | .266 | .115 | 5.377 | 1 | .020 | 1.304 | 1.042 | 1.633 |
| Gender- Female | .008 | .124 | .004 | 1 | .948 | 1.008 | .791 | 1.285 |
| Education- Literate | .166 | .163 | 1.038 | 1 | .308 | 1.180 | .858 | 1.624 |
| Age- > 60 years |  |  | 2.448 | 3 | .485 |  |  |  |
| Age- Below 5 years | .467 | .681 | .470 | 1 | .493 | 1.595 | .420 | 6.062 |
| Age- 5-15 years | .232 | .257 | .817 | 1 | .366 | 1.261 | .763 | 2.085 |
| Age- 16-60 years | .271 | .178 | 2.309 | 1 | .129 | 1.311 | .924 | 1.860 |
| Disease- Other diseases |  |  | 49.373 | 3 | .000 |  |  |  |
| Disease- Communicable and MCH related | .297 | .155 | 3.701 | 1 | .054 | 1.346 | .994 | 1.823 |
| Disease- Non-communicable | 1.114 | .173 | 41.586 | 1 | .000 | 3.047 | 2.172 | 4.274 |
| Disease- Injuries | .779 | .249 | 9.821 | 1 | .002 | 2.180 | 1.339 | 3.548 |
| Wealth Quintile- Poorest |  |  | 172.278 | 4 | .000 |  |  |  |
| Wealth Quintile- Poor | 2.869 | .249 | 132.342 | 1 | .000 | 17.619 | 10.807 | 28.726 |
| Wealth Quintile- Middle | 1.658 | .192 | 74.954 | 1 | .000 | 5.251 | 3.607 | 7.644 |
| Wealth Quintile- Rich | 1.525 | .171 | 79.735 | 1 | .000 | 4.594 | 3.287 | 6.419 |
| Wealth Quintile- Richest | 1.072 | .153 | 49.377 | 1 | .000 | 2.921 | 2.166 | 3.938 |
| Constant | -3.472 | .297 | 136.321 | 1 | .000 | .031 |  |  |

**Model 5**

| Factors | B | S.E. | Wald | df | Sig. | Exp(B) | 95% C.I. for EXP(B) | |
| --- | --- | --- | --- | --- | --- | --- | --- | --- |
|  |  |  |  |  |  |  | Lower | Upper |
| Insured with RSBY | .541 | .455 | 1.409 | 1 | .235 | 1.717 | .703 | 4.191 |
| Caste- General category |  |  | 5.516 | 3 | .138 |  |  |  |
| Caste- Schedule caste | .591 | .419 | 1.985 | 1 | .159 | 1.806 | .794 | 4.109 |
| Caste- Schedule Tribe | .890 | .479 | 3.453 | 1 | .063 | 2.436 | .952 | 6.230 |
| Caste- Other Backward class | -.018 | .375 | .002 | 1 | .961 | .982 | .471 | 2.046 |
| Religion- Others |  |  | 5.911 | 2 | .052 |  |  |  |
| Religion- Hindu | 1.709 | .703 | 5.911 | 1 | .015 | 5.524 | 1.393 | 21.913 |
| Religion- Muslim | -21.903 | 40192.970 | .000 | 1 | 1.000 | .000 | 0.000 |  |
| Household size- Less than 5 members | .367 | .290 | 1.598 | 1 | .206 | 1.443 | .817 | 2.549 |
| Gender- Female | -.188 | .304 | .383 | 1 | .536 | .828 | .457 | 1.503 |
| Education- Literate | -.271 | .345 | .616 | 1 | .433 | .763 | .388 | 1.500 |
| Age- > 60 years |  |  | 1.683 | 2 | .431 |  |  |  |
| Age- Below 5 years | .905 | .702 | 1.660 | 1 | .198 | 2.471 | .624 | 9.787 |
| Age- 15-60 years | .513 | .565 | .826 | 1 | .364 | 1.671 | .552 | 5.057 |
| Disease- Other diseases |  |  | 2.534 | 3 | .469 |  |  |  |
| Disease- Communicable and MCH related | .605 | .457 | 1.747 | 1 | .186 | 1.830 | .747 | 4.486 |
| Disease- Non-communicable | .303 | .474 | .408 | 1 | .523 | 1.354 | .535 | 3.427 |
| Disease- Injuries | -.011 | .670 | .000 | 1 | .987 | .989 | .266 | 3.676 |
| Wealth Quintile- Poorest |  |  | 4.184 | 4 | .382 |  |  |  |
| Wealth Quintile- Poor | -.177 | .504 | .123 | 1 | .725 | .838 | .312 | 2.251 |
| Wealth Quintile- Middle | -.269 | .491 | .301 | 1 | .584 | .764 | .292 | 1.999 |
| Wealth Quintile- Rich | -1.015 | .584 | 3.019 | 1 | .082 | .363 | .115 | 1.139 |
| Wealth Quintile- Richest | -.001 | .519 | .000 | 1 | .998 | .999 | .361 | 2.759 |
| State- Uttar Pradesh |  |  | 2.443 | 2 | .295 |  |  |  |
| State- Haryana | .280 | .471 | .354 | 1 | .552 | 1.323 | .526 | 3.333 |
| State- Gujarat | -.371 | .415 | .800 | 1 | .371 | .690 | .306 | 1.555 |
| Constant | -1.952 | .863 | 5.119 | 1 | .024 | .142 |  |  |

**Model 6**

| Factors | B | S.E. | Wald | df | Sig. | Exp(B) | 95% C.I. for EXP(B) | |
| --- | --- | --- | --- | --- | --- | --- | --- | --- |
|  |  |  |  |  |  |  | Lower | Upper |
| Insured | .028 | .142 | .039 | 1 | .844 | 1.028 | .779 | 1.358 |
| Caste- General category |  |  | 6.485 | 3 | .090 |  |  |  |
| Caste- Schedule caste | .288 | .176 | 2.662 | 1 | .103 | 1.334 | .944 | 1.885 |
| Caste- Schedule Tribe | -.304 | .244 | 1.550 | 1 | .213 | .738 | .457 | 1.191 |
| Caste- Other Backward class | .158 | .138 | 1.312 | 1 | .252 | 1.171 | .894 | 1.536 |
| Religion- Others |  |  | 8.566 | 4 | .073 |  |  |  |
| Religion- Hindu | -.713 | .249 | 8.203 | 1 | .004 | .490 | .301 | .798 |
| Religion- Muslim | 22.164 | 40192.969 | .000 | 1 | 1.000 | 4225679646.344 | 0.000 |  |
| Religion- Christian | -.473 | .737 | .412 | 1 | .521 | .623 | .147 | 2.642 |
| Caste- General category | -19.886 | 23148.561 | .000 | 1 | .999 | .000 | 0.000 |  |
| Household size- Less than 5 members | -.676 | .120 | 31.533 | 1 | .000 | .509 | .402 | .644 |
| Gender- Female | -.131 | .125 | 1.086 | 1 | .297 | .877 | .686 | 1.122 |
| Education- Literate | .112 | .152 | .542 | 1 | .462 | 1.118 | .831 | 1.505 |
| Age- > 60 years |  |  | 3.540 | 3 | .316 |  |  |  |
| Age- Below 5 years | .160 | .695 | .053 | 1 | .817 | 1.174 | .301 | 4.579 |
| Age- 5-15 years | -.481 | .270 | 3.181 | 1 | .075 | .618 | .365 | 1.049 |
| Age- 16-60 years | -.114 | .173 | .433 | 1 | .510 | .893 | .636 | 1.252 |
| Health Facility Type- Public |  |  | 102.048 | 2 | .000 |  |  |  |
| Health Facility Type- Private | 1.569 | .165 | 90.912 | 1 | .000 | 4.801 | 3.478 | 6.629 |
| Health Facility Type- NGO/Charitable/Others | .160 | .361 | .195 | 1 | .659 | 1.173 | .578 | 2.381 |
| Admission Days- <2days |  |  | 182.555 | 2 | .000 |  |  |  |
| Admission Days- 2-5 days | .828 | .180 | 21.286 | 1 | .000 | 2.289 | 1.610 | 3.255 |
| Admission Days- >5days | 2.170 | .181 | 144.280 | 1 | .000 | 8.754 | 6.144 | 12.473 |
| Disease- Other diseases |  |  | 51.490 | 3 | .000 |  |  |  |
| Disease- Communicable and MCH related | -.178 | .178 | 1.000 | 1 | .317 | .837 | .590 | 1.187 |
| Disease- Non-communicable | .635 | .174 | 13.224 | 1 | .000 | 1.886 | 1.340 | 2.655 |
| Disease- Injuries | .998 | .235 | 18.046 | 1 | .000 | 2.714 | 1.712 | 4.302 |
| Wealth Quintile- Poorest |  |  | 184.537 | 4 | .000 |  |  |  |
| Wealth Quintile- Poor | 2.670 | .213 | 157.780 | 1 | .000 | 14.435 | 9.517 | 21.894 |
| Wealth Quintile- Middle | 1.800 | .195 | 85.612 | 1 | .000 | 6.049 | 4.131 | 8.856 |
| Wealth Quintile- Rich | 1.443 | .184 | 61.392 | 1 | .000 | 4.234 | 2.951 | 6.075 |
| Wealth Quintile- Richest | .704 | .185 | 14.496 | 1 | .000 | 2.022 | 1.407 | 2.905 |
| State- Uttar Pradesh |  |  | 3.821 | 2 | .148 |  |  |  |
| State- Haryana | -.170 | .154 | 1.227 | 1 | .268 | .843 | .624 | 1.140 |
| State- Gujarat | .133 | .156 | .732 | 1 | .392 | 1.143 | .842 | 1.552 |
| Constant | -4.462 | .357 | 156.337 | 1 | .000 | .012 |  |  |

**Model 7**

| Factors | B | S.E. | Wald | df | Sig. | Exp(B) | 95% C.I. for EXP(B) | |
| --- | --- | --- | --- | --- | --- | --- | --- | --- |
|  |  |  |  |  |  |  | Lower | Upper |
| Insured with RSBY | .903 | .390 | 5.365 | 1 | .021 | 2.466 | 1.149 | 5.294 |
| Caste- General category |  |  | 4.276 | 3 | .233 |  |  |  |
| Caste- Schedule caste | -.499 | .404 | 1.527 | 1 | .217 | .607 | .275 | 1.340 |
| Caste- Schedule Tribe | -.858 | .531 | 2.613 | 1 | .106 | .424 | .150 | 1.200 |
| Caste- Other Backward class | -.598 | .339 | 3.118 | 1 | .077 | .550 | .283 | 1.068 |
| Religion- Others |  |  | .190 | 2 | .909 |  |  |  |
| Religion- Hindu | -.427 | .979 | .190 | 1 | .663 | .652 | .096 | 4.445 |
| Religion- Muslim | 21.828 | 40192.969 | .000 | 1 | 1.000 | 3018019570.105 | 0.000 |  |
| Religion- Christian | -.941 | .282 | 11.148 | 1 | .001 | .390 | .224 | .678 |
| Caste- General category | -.015 | .283 | .003 | 1 | .959 | .985 | .566 | 1.716 |
| Household size- Less than 5 members | .247 | .346 | .508 | 1 | .476 | 1.280 | .649 | 2.522 |
| Age- > 60 years |  |  | 7.740 | 3 | .052 |  |  |  |
| Age- Below 5 years | -18.844 | 21419.480 | .000 | 1 | .999 | .000 | 0.000 |  |
| Age- 5-15 years | -1.715 | .633 | 7.350 | 1 | .007 | .180 | .052 | .622 |
| Disease- Other diseases | -.736 | .372 | 3.911 | 1 | .048 | .479 | .231 | .993 |
| Disease- Communicable and MCH related |  |  | 11.989 | 3 | .007 |  |  |  |
| Disease- Non-communicable | -.468 | .402 | 1.360 | 1 | .244 | .626 | .285 | 1.376 |
| Disease- Injuries | .087 | .395 | .048 | 1 | .827 | 1.090 | .503 | 2.365 |
| Disease- Other diseases | 1.209 | .552 | 4.799 | 1 | .028 | 3.350 | 1.136 | 9.883 |
| Wealth Quintile- Poorest |  |  | 39.783 | 4 | .000 |  |  |  |
| Wealth Quintile- Poor | 2.872 | .499 | 33.078 | 1 | .000 | 17.671 | 6.641 | 47.024 |
| Wealth Quintile- Middle | 1.513 | .416 | 13.214 | 1 | .000 | 4.541 | 2.008 | 10.267 |
| Wealth Quintile- Rich | 1.013 | .391 | 6.720 | 1 | .010 | 2.754 | 1.280 | 5.924 |
| Wealth Quintile- Richest | .209 | .409 | .263 | 1 | .608 | 1.233 | .554 | 2.746 |
| State- Uttar Pradesh |  |  | 1.878 | 2 | .391 |  |  |  |
| State- Haryana | -.631 | .478 | 1.745 | 1 | .187 | .532 | .209 | 1.357 |
| State- Gujarat | -.478 | .407 | 1.383 | 1 | .240 | .620 | .279 | 1.375 |
| Health Facility Type- Public |  |  | 28.732 | 2 | .000 |  |  |  |
| Health Facility Type- Private | 1.999 | .409 | 23.919 | 1 | .000 | 7.384 | 3.314 | 16.452 |
| Health Facility Type- NGO/Charitable/Others | -.055 | .900 | .004 | 1 | .951 | .946 | .162 | 5.527 |
| Admission Days- <2days |  |  | 25.836 | 2 | .000 |  |  |  |
| Admission Days- 2-5 days | .340 | .391 | .754 | 1 | .385 | 1.405 | .652 | 3.025 |
| Admission Days- >5days | 1.587 | .382 | 17.275 | 1 | .000 | 4.890 | 2.313 | 10.336 |
| Constant | -2.803 | .769 | 13.272 | 1 | .000 | .061 |  |  |

**Model 8**

| Factors | B | S.E. | Wald | df | Sig. | Exp(B) | 95% C.I. for EXP(B) | |
| --- | --- | --- | --- | --- | --- | --- | --- | --- |
|  |  |  |  |  |  |  | Lower | Upper |
| Insured with RSBY | 1.008 | .658 | 2.346 | 1 | .126 | 2.741 | .754 | 9.960 |
| Caste- General category |  |  | 4.346 | 3 | .226 |  |  |  |
| Caste- Schedule caste | -.758 | .544 | 1.940 | 1 | .164 | .469 | .161 | 1.362 |
| Caste- Schedule Tribe | -.871 | .617 | 1.994 | 1 | .158 | .418 | .125 | 1.402 |
| Caste- Other Backward class | -.924 | .462 | 3.994 | 1 | .046 | .397 | .160 | .982 |
| Religion- Others |  |  | .058 | 2 | .971 |  |  |  |
| Religion- Hindu | .296 | 1.229 | .058 | 1 | .810 | 1.345 | .121 | 14.946 |
| Religion- Muslim | 21.168 | 40192.969 | .000 | 1 | 1.000 | 1559720098.430 | 0.000 |  |
| Household size- Less than 5 members | -.804 | .366 | 4.813 | 1 | .028 | .448 | .218 | .918 |
| Gender- Female | -.334 | .375 | .792 | 1 | .373 | .716 | .343 | 1.494 |
| Education- Literate | .363 | .390 | .868 | 1 | .352 | 1.438 | .670 | 3.084 |
| Age- > 60 years |  |  | 8.382 | 2 | .015 |  |  |  |
| Age- Below 5 years | -2.394 | .865 | 7.664 | 1 | .006 | .091 | .017 | .497 |
| Age- 5-15 years | -1.471 | .594 | 6.138 | 1 | .013 | .230 | .072 | .735 |
| Disease- Other diseases |  |  | 3.737 | 3 | .291 |  |  |  |
| Disease- Communicable and MCH related | -.395 | .570 | .480 | 1 | .488 | .674 | .220 | 2.059 |
| Disease- Non-communicable | -.146 | .583 | .062 | 1 | .803 | .864 | .276 | 2.712 |
| Disease- Injuries | .861 | .784 | 1.206 | 1 | .272 | 2.366 | .509 | 10.995 |
| Wealth Quintile- Poorest |  |  | 26.502 | 4 | .000 |  |  |  |
| Wealth Quintile- Poor | 2.839 | .664 | 18.276 | 1 | .000 | 17.106 | 4.654 | 62.881 |
| Wealth Quintile- Middle | 1.370 | .594 | 5.323 | 1 | .021 | 3.933 | 1.229 | 12.590 |
| Wealth Quintile- Rich | 1.231 | .622 | 3.924 | 1 | .048 | 3.426 | 1.013 | 11.584 |
| Wealth Quintile- Richest | -.207 | .648 | .102 | 1 | .749 | .813 | .228 | 2.897 |
| State- Uttar Pradesh |  |  | 1.730 | 2 | .421 |  |  |  |
| State- Haryana | -.416 | .621 | .448 | 1 | .503 | .660 | .195 | 2.228 |
| State- Gujarat | -.588 | .450 | 1.709 | 1 | .191 | .555 | .230 | 1.341 |
| Health Facility Type- Public |  |  | 24.029 | 2 | .000 |  |  |  |
| Health Facility Type- Private | 2.428 | .522 | 21.601 | 1 | .000 | 11.332 | 4.071 | 31.542 |
| Health Facility Type- NGO/Charitable/Others | .357 | 1.239 | .083 | 1 | .773 | 1.430 | .126 | 16.199 |
| Admission Days- <2days |  |  | 16.818 | 2 | .000 |  |  |  |
| Admission Days- 2-5 days | .037 | .500 | .005 | 1 | .942 | 1.037 | .390 | 2.762 |
| Admission Days- >5days | 1.489 | .491 | 9.214 | 1 | .002 | 4.433 | 1.695 | 11.596 |
| Constant | -2.079 | 1.218 | 2.915 | 1 | .088 | .125 |  |  |
